# Conceptual and methodological challenges to measuring political commitment to respond to HIV

**DOI:** 10.1186/1758-2652-14-S2-S5

**Published:** 2011-09-27

**Authors:** Ashley M Fox, Allison B Goldberg, Radhika J Gore, Till Bärnighausen

**Affiliations:** 1 Division of Health Policy and Administration, Yale School of Public Health, 60 College Street, New Haven, CT, USA; 2 Department of Sociomedical Sciences, Mailman School of Public Health, Columbia University, 722 W168th Street, New York, NY, USA; 3 Department of Global Health and Population, Harvard School of Public Health, Boston, MA, USA; 4 Africa Centre for Health and Population Studies, University of KwaZulu-Natal, Mtubatuba, South Africa

## Abstract

**Background:**

Researchers have long recognized the importance of a central government’s political “commitment” in order to mount an effective response to HIV. The concept of political commitment remains ill-defined, however, and little guidance has been given on how to measure this construct and its relationship with HIV-related outcomes. Several countries have experienced declines in HIV infection rates, but conceptual difficulties arise in linking these declines to political commitment as opposed to underlying social and behavioural factors.

**Methods:**

This paper first presents a critical review of the literature on existing efforts to conceptualize and measure political commitment to respond to HIV and the linkages between political commitment and HIV-related outcomes. Based on the elements identified in this review, the paper then develops and presents a framework to assist researchers in making choices about how to assess a government's level of political commitment to respond to HIV and how to link political commitment to HIV-related outcomes.

**Results:**

The review of existing studies identifies three components of commitment (expressed, institutional and budgetary commitment) as different dimensions along which commitment can be measured. The review also identifies normative and ideological aspects of commitment and a set of variables that mediate and moderate political commitment that need to be accounted for in order to draw valid inferences about the relationship between political commitment and HIV-related outcomes. The framework summarizes a set of steps that researchers can follow in order to assess a government's level of commitment to respond to HIV and suggests ways to apply the framework to country cases.

**Conclusions:**

Whereas existing studies have adopted a limited and often ambiguous conception of political commitment, we argue that conceiving of political commitment along a greater number of dimensions will allow researchers to draw a more complete picture of political commitment to respond to HIV that avoids making invalid inferences about the relationship between political commitment and HIV outcomes.

## Background

Researchers have long recognized the importance of a central government's political “commitment” in order to mount an effective response to HIV [1-12]. In spite of this recognition, empirical research examining the effects of political commitment on HIV outcomes remains sparse [13], and the concept of political commitment, also sometimes referred to as “political will”, ill-defined [13,15,16]. Indeed, a government’s political commitment to respond to HIV is often judged loosely in terms of countries’ “reputations” for good or bad leadership on HIV with little empirical justification [11,12]. Without clarity about how to assess a central government’s political commitment to respond to HIV, countries cannot be held accountable for inadequately responding to this epidemic, nor can committed countries provide lessons about effective policy approaches to other countries.

Lack of conceptual clarity over how to assess political commitment may furthermore lead researchers to draw invalid inferences about the relationship between commitment and outcomes. As Youde (2007) notes, researchers have tended to infer varying levels of political commitment based on HIV outcomes [10]. Countries with continued high prevalence rates are blamed for their failure to adequately respond, whereas countries that have maintained low prevalence rates are praised for keeping their epidemics under control. This approach to measuring political commitment is problematic because commitment is measured in terms of a successful or unsuccessful outcome, instead of being measured separately from the outcome of interest [10]. In order to assign causal significance to political commitment, it is necessary to establish a relevant counterfactual: what would the HIV infection rate in countries with strong commitment have been if their commitment had, contrary to fact, been weak?

A number of recent “success” cases in HIV reduction draw into question how political commitment contributes to HIV outcomes. Zimbabwe, Kenya and Haiti have recently experienced declines in HIV prevalence that can be explained by parallel changes in behaviour [17-20]. Yet, unlike early country examples, such as Uganda and Thailand where declines could plausibly be linked to explicit government policy responses [21,22], these countries are not distinguished for clearly having strong political commitment targeting prevention and behaviour change. By contrast, Botswana is considered successful in developing effective treatment policies, but has not been deemed successful at preventing new infections; this is what Swidler (2006) refers to as the “Botswana paradox” [23]. Without a clear understanding of how political commitment contributes to HIV outcomes, debate will continue about the degree to which declines can be attributed to policy responses versus the natural dynamics of the epidemic, or other reasons, such as social changes and grassroots responses unrelated to government commitment [17,24].

Although commonly invoked in the public health literature, within political science, the concept of political commitment has not been well explored. Political scientists consider leadership characteristics to be idiosyncratic [25], to lack predictive power [26], and to fail to account for long-term differences in policy trajectories over time [27,28]. Students of political science instead look to more durable reasons for policy differences across countries, such as variations in political institutions [27-30], political culture [31,32] and trends in competitive politics [33,34]. Policies are believed to come onto the national agenda not because of the strong initiative of individual leaders exclusively, but rather because of a confluence of several factors coming together at the right moment, including the existence of a ready-made policy solution, which addresses a persistent problem and the opening of a political window of opportunity as a result of elections or the advent of a crisis [35]. In this way, the commitment of leaders is insufficient to assure attention to an issue if these other factors are not present [35].

In democratic and multi-party systems, the opinions of leaders are believed to reflect the will of the public, and voters can either hold governments accountable for their policy responses through voting them out of office or can prospectively elect a leader who pledges her commitment to a particular policy platform [36]. International norms and policy ideas that are advocated by international institutions like the World Bank are also likely to influence leaders’ policy choices [34,38]. On the other hand, research in other policy domains has shown that leaders do not simply follow the preferences of their constituents in formulating policy; rather, the content of policies differ between leaders who are “true believers” in a policy compared with leaders who are “converts” and follow popular opinion or pressure from international institutions [34]. True believers implement policy as intended by policy designers, whereas converts implement policy in a manner that is more in line with their actual beliefs. Thus, even leaders who adopt the same policies in name may implement them very differently. Politicians can also frame the reasons for their policy choices differently to make them more compelling to their constituents [39]. One challenge in measuring political commitment from a political science perspective is therefore the question of how to know whether a leader is genuinely committed to a policy platform or is feigning commitment for strategic reasons or under pressure from external forces and may therefore be more likely to renege on commitments or implement them in a self-defeating manner. Leaders have an incentive to support popular policies or policies tied to continued international assistance even if ideologically they do not agree with them.

Furthermore, with regular turnover of leadership in electoral democracies, there is also the risk that policies established under one administration will be reversed if there is a change in leadership. The question thus becomes one of “credible” commitment, i.e., can actors be assured that the government will commit in a way that would make later policy reversals highly unlikely [40-42]? The pronouncements of the state and its concrete actions constitute its signal of credibility, i.e., of a “credible commitment” whereby actors expect the state to abide by agreed upon or expressed policies and not renege or arbitrarily exercise discretionary power [41]. The crucial question in assessing the credibility of commitment is therefore whether constituents and advocates can identify mechanisms that effectively tie the hands of governments making policy reversal difficult.

As this brief review illustrates, because political science research is primarily interested in explaining political phenomena rather than health outcomes, this body of research has tended to focus on explaining political commitment rather than the effects of political commitment on policy outcomes. This line of research is less useful to researchers in the health field who wish to assess the latter relationship. Consequently, the difficulty that health researchers experience in measuring political commitment can be partly attributed to a dearth of existing theory on political commitment that links it to outcomes.

These constraints have left a knowledge gap in the field on how political commitment translates into effective government action on HIV outcomes. A number of indicators of HIV-specific political commitment are available, which could be used to study the effect of commitment on government action and HIV outcomes, including the AIDS Program Effort Index [43], the United Nations General Assembly Special Session on HIV/AIDS (UNGASS) Declaration of Commitment Indicators [44], and the AIDS Policy Aggressiveness Indicators [45]. Despite their potential, these measures have only rarely been used to assess the effect of commitment on outcomes.

## Methods

Given the gap between the importance that is ascribed to political commitment and what is actually known about the contribution of political commitment to HIV outcomes, it is important to establish a framework to assist researchers in conceptualizing and measuring political commitment in a way that is empirically testable. To that end, this article first critically reviews existing efforts to assess political commitment to respond to HIV and describes and critiques three major components along which commitment has been conceptualized in the literature: (1) expressed commitment; (2) institutional commitment; and (3) budgetary commitment.

The paper then identifies normative and ideological aspects of government responses to HIV that cut across these three dimensions that further complicate the measurement of commitment and suggests ways that researchers can approach the measurement of these aspects to produce clear research questions. The article additionally argues that it is necessary to control in the analysis for factors that influence countries’ ability to respond to this epidemic in order to draw inferences about the relationship between political commitment and HIV outcomes.

Based on this review and political science theory on political commitment, the paper then develops a conceptual framework to assist researchers in assessing a government’s level of political commitment to respond to HIV, and provides examples of how the framework can be used to draw valid inferences about the effect of political commitment on HIV-related outcomes. Although this framework is developed with HIV in mind, we encourage researchers to test the robustness of this conceptualization of political commitment to assess its impact on other public health threats.

## Results

### Conceptualizing political commitment to respond to HIV

Before attributions of commitment and success can be assigned to government policy responses to HIV, researchers must first arrive at a common definition of what constitutes “political commitment”. Different authors have defined and operationalized political commitment in different ways. Some researchers have judged political commitment based on what governments say rather than what they do [1,4,10,14,47-49]. Others have focused on the presence of institutional structures that enable a response to this disease [50,51] or they have emphasized how much governments invest in HIV programmes as a measure of commitment [11,45]. Still others have examined HIV service delivery outcomes, such as access to antiretrovirals (ARVs) and prevention of mother to child transmission (PMTCT) programmes, controlling for country-level resources in attempts to parse out governmental effort to respond to HIV from its ability to respond to this disease [11,12].

Examining each of these components in isolation has the potential to lead to an incomplete picture of government commitment, as well as to invalid inferences about the relationship between political commitment and HIV policy outcomes. The following section summarizes existing literature on political commitment to respond to HIV, grouping studies according to their conceptualizations of commitment and the factors that affect countries’ level of commitment.

### Expressed commitment

Based on often implicit criteria, researchers have tended to infer a government’s commitment to respond to HIV according to how often and early key government leaders make public statements about HIV [11]. Leaders who are willing to address HIV openly, candidly and in a timely manner are generally treated as “committed” to responding to HIV [10,48]. For example, researchers have interpreted President Yoweri Museveni’s willingness to speak openly about HIV in Uganda as a sign of his commitment to controlling the epidemic [7,10,48]. On the other hand, former US President Ronald Reagan’s unwillingness to speak openly about HIV in the early stages of the epidemic was taken as a sign of a lack of commitment to fighting the disease [46], as was former South African President Thabo Mbeki’s equivocation on HIV virology [4,10,14,49,52]. Expressed commitment has also been conceptualized as “symbolic politics”: policy makers can use language and images as symbols to set health policy agendas and to change conceptions of issues, such as the causes and consequences of HIV [16]. Studies have measured verbal commitments by analyzing speeches of key leadership [45,49,52], discourse analysis of media texts [53], and assessing the general tone and tenor of the response [1,4,10,14,48].

### Institutional commitment

In addition to verbal expressions of commitment, a critical step in many countries is setting up the basic “institutions” or bureaucratic infrastructure needed to develop a response. The timing of each of these developments has been consequential in assessing a government’s level of commitment. For example, Gauri and Lieberman (2006) interpret “when” national AIDS commissions were first introduced in Brazil and South Africa (early versus late in the course of the epidemic) as an indicator of a proactive response in the former, and a reactive response in the latter [25]. By building up infrastructure and procedures that are hard to undo once established, institutional commitment goes beyond mere proclamations of commitment, creating mechanisms that credibly “lock in” the state’s response [25-30]. These HIV-related institutions – formal and informal “rules of the game” [54] – are difficult to undo because they come about through political processes, including negotiations, agreements and the mobilization of human and financial resources.

In Brazil, for example, Nunn (2009) argues that AIDS treatment institutions introduced early in the response to HIV, including formal and informal programmes and policies committing the state to AIDS treatment, set the country on a path towards a commitment to treatment access [51]. Likewise, the Ryan White legislation in the US has set up structures and expectations that have made the reversal of this legislation and HIV-specific funding difficult to undo [46].

Measuring the ways that governments translate their verbal commitments to fighting HIV into infrastructure, policies and procedures (i.e., institutions) therefore constitutes an additional component of political commitment that researchers can measure to assess a government’s overall level of commitment to respond to HIV. The existence of HIV institutions in a country has been measured in terms of the establishment of a national AIDS commission [25,50], securing a safe blood supply [56], developing an HIV sero-surveillance system [56], and adopting policies aimed at prevention, treatment, care and support [25,50,51,56].

### Budgetary commitment

Public pronouncements and policy enactments alone may not provide a complete picture of governmental commitment to respond to HIV without the tangible resource allocations to support these pronouncements and policies. Commitment is signalled not only as a “promise” about the future (e.g., a political leader making promises in a media announcement, which may be a losing battle or an empty proposal from the beginning), but also as the matching up of words with action, something that can often only be assessed in hindsight, i.e., by comparing what government said and what it ultimately did. Lieberman (2009), for example, disagrees that leadership qualities, which he views as idiosyncratic, should be used as a measure of political commitment [45]. Instead, he measures countries’ responses in terms of their relative resource allocations, which he views as a more objective measure of commitment. Assessing commitment as resource allocation allows for an evaluation of whether commitment lives up to verbal rhetoric and institutional commitments [23].

Expenditure actually dispersed rather than pledged provides a more concrete measure of commitment, but the exact measurement of resource allocations (e.g., total expenditure on HIV per capita, the percentage of the country’s total budget, etc.) should depend on the research question under investigation and the type of study (i.e., cross-country comparison vs. single country case study). Some studies have assessed commitment not only in terms of resource allocations, but also in terms of service delivery or the availability of antiretroviral therapy (ART) and PMTCT therapies [11,12]. This approach is problematic since HIV-related services are best thought of as outputs of political commitment, not measures of commitment themselves (see section on outputs and outcomes).

### Normative and evidence-based aspects of political commitment

In addition to objective measures of political commitment, such as the number of public pronouncements made by government leaders, the creation (or not) of institutions to address HIV, and public expenditures towards HIV, a government’s commitment to respond to HIV has also been assessed along normative and ideological lines: the ethical and human rights aspects of government commitment to respond to HIV and the extent to which the political response is grounded in scientific evidence and international best practices [35-63]. These normative and evidence-based aspects of commitment cut across the three dimensions of political commitment and require researchers’ discretion over how they are ultimately coded with regards to commitment.

#### Normative aspects of commitment

Perhaps more than for other diseases, the balance between the rights of people living with HIV and public health prerogatives has been a central tension addressed by HIV researchers [57]. HIV has been described as “exceptional” for its focus on the protection of the rights of individuals living with the disease [55]. Though there has been some debate about whether the rights-based approach that characterized the response in the developed countries with HIV epidemics that are concentrated in specific risk groups is valid in developing countries with generalized epidemics, researchers and human rights groups routinely assess governments in terms of the degree to which their policies constrain the rights of people living with HIV and marginalized groups that are disproportionately affected by the epidemic [57-61,63,64].

Existing research on political commitment and HIV has treated these normative elements of a government’s response in various ways. Baldwin (2005) integrates the issue of rights-based versus rights-constraining responses directly into his research question by asking why developed countries have differed in their responses in this respect [56]. He distinguishes between countries that adopted harsh or coercive HIV policies (e.g., quarantine, compulsory institutionalization, and forcible treatment) and those that adopted a voluntarist approach (e.g., education, counselling, and voluntary behavioural change) in their responses.

In Baldwin’s framework, countries that adopt either approach could be construed as committed to responding to HIV, although through different methods. A country can commit strongly to enforcing coercive policies, reinforcing the need for these measures through public messages, creating institutions to enforce coercive policies through quarantine and forced treatment protocols, and dedicating public funds towards this end, just as another country could do the same for voluntarist policies. Baldwin’s framework, therefore, is agnostic to the type of approach adopted; rather, he seeks to understand why states have adopted one approach or the other.

Rather than being agnostic to the normative dimensions of the response, Lieberman specifically defines the “policy aggressiveness” of a country as the degree to which country policies comport with a “Geneva Consensus” approach to responding to HIV [45]. In his definition, the “Geneva Consensus” refers to the recognized international standards, including human rights, put forth by the World Health Organization (WHO) and UNAIDS, the major international institutions in charge of overseeing the global response to HIV [45]. Under Lieber-man’s assessment, a state that violates international human rights standards would not be considered to be committed to responding to HIV even if the state expresses its commitment, develops institutions towards its policy response, and allots funds in support of these institutions.

Overall, the normative aspects of HIV policy responses raise questions about how to judge a government’s level of commitment to respond to HIV. It is not immediately clear how to categorize these normative aspects of a response as signals of commitment, and researchers’ discretion is necessary to decide how to define commitment along these lines. Should a government that aggressively commits to a response that violates the rights of individuals living with HIV be considered as “committed” to responding to HIV? Cuba, for example, has been criticized for its use of quarantine measures to prevent the further spread of HIV in the early years in the epidemic, although infection rates in Cuba remain the lowest in the Caribbean region [65].

Likewise, some researchers have questioned whether Botswana’s routine opt-out testing is ethically sound, although the government is committed to this measure, which it views as necessary to increase testing rates and enhance prevention efforts [58]. In Zimbabwe, president Robert Mugabe’s homophobic remarks portraying HIV infection as a Western disease that is out of step with African traditions, has been interpreted as evidence of the government’s lack of commitment to fighting the epidemic, even though HIV was being publicly discussed [7]. These examples illustrate that a government can be committed to a response deemed unethical by the international community. The Cuba example further illustrates that there is no necessary relationship between the effectiveness of an HIV response and whether or not that response respects human rights. By measuring commitment exclusively in terms of what is regarded as acceptable by some normative standards, researchers might draw the wrong conclusions regarding the effect of government commitment on HIV-related outcomes.

#### Evidence-based aspects of commitment

Related to the normative dimensions of measuring government commitment is the question of how to judge which types of policy responses - those grounded in empirical evidence or more experimental policies conforming with ideological preferences - should be considered to represent government commitment to respond to HIV. A number of studies have defined political commitment in terms of rhetoric and policies supportive of internationally accepted scientific evidence as opposed to those lacking scientific support [4,10,52,59-61].

For example, the success of Uganda relative to South Africa in combating HIV has been attributed to the fact that medical evidence about HIV guided Uganda’s response, whereas some of South Africa’s leaders adhered to dissident scientific viewpoints [4,10,48,49]. Uganda’s more recent turn from emphasizing fidelity to emphasizing abstinence has been viewed as being ideologically driven by donors’ prioritization of abstinence programmes [61,62]. A similar example is the debate over sex education in the US under the administration of George W. Bush, which has been described as a struggle between scientific consensus and conservative religious ideology [61,63]; yet, George W. Bush has committed more in terms of budgetary resources to international HIV efforts than any of the previous U.S. presidents. The refusal of many countries, including certain US states and Russia, to adopt harm-reduction policies through needle-exchange programmes constitutes another example of the tension between scientific evidence and ideological aversions to certain policy options [66,67]. As with normative standards, Lieberman explicitly defines political commitment or “policy aggressiveness” in terms of meeting Geneva Consensus scientific standards.

By contrast, Epstein (2007) describes how the international community ignored evidence on the role of male circumcision in reducing the spread of HIV in Africa, and continued to promote condom use as a primary prevention strategy despite lack of evidence on its effectiveness [62]. Because scientific evidence is deeply contested and evidence continues to accrue, theories that were once on the fringe, such as male circumcision, have become accepted as best practices and adopted by policy makers, whereas standards that are now considered ineffectual, such as the exclusive promotion of condom use in Africa, were at one time the accepted dogma. By measuring commitment exclusively in terms of commitment to evidence-based policies, researchers run the risk of excluding policies that might actually be effective, but that have not yet been proven effective. Because political logic and empirical evidence are often in tension, researchers, who employ a measure of commitment agnostic to adherence with best practices, may arrive at very different conclusions from researchers, who assess commitment in terms of policies’ comporting with science.

In addition, a possible unintended consequence of measuring political commitment in terms of compliance with international standards is that countries might view adhering to global best practices under the threat of being criticized for a lack of commitment as a tool of international institutions that impedes their autonomy. Due to this international pressure, leaders who are concerned about international reputation might be less willing to experiment with cutting-edge policies that lack sufficient empirical support but are nonetheless effective. Alternatively, policymakers who wish to demonstrate their independence from international pressure may support fringe policies in spite of the risk these policies pose. In general, policy makers must often make policy decisions in the absence of adequate information and with equal attention to the reaction of their constituents as to the effectiveness of their policy choices [36].

#### Measuring normative and evidence-based aspects of a response

Given the conceptual challenges posed by the normative and evidence-based elements of a government’s response, researchers should understand the trade-offs of defining commitment in one way or another, but should at minimum be explicit in how they are defining commitment. If researchers adopt approaches agnostic to the normative and evidence-based aspects of the response, they may choose normatively and ideologically ambiguous measures of political commitment, e.g., number of mentions of HIV in speeches (regardless of tenor), number of HIV-related policies adopted (regardless of content), and total expenditures on HIV (regardless of purpose).

Alternatively, to assess commitment in terms of correspondence with rights-based policy guidelines, governments can be measured as committing to a rights-based response verbally through public pronouncements that are supportive of the rights of affected groups, institutionally through laws and legislation explicitly protecting the rights of affected groups, and in terms of budget allocations to meet the explicit needs of affected groups. Likewise, should researchers decide to measure commitment exclusively in terms of commitment to evidence-based policy, the correspondence of commitment with medical evidence can be measured in terms of what governments say (e.g., questioning whether HIV causes AIDS), whether governments adopt policies that are evidence-based (e.g., abstinence-only or comprehensive sex education), and whether they allocate resources towards evidence-based treatments and programmes (e.g., towards ART rather than towards “therapies” of unproven effectiveness).

In sum, based on how the term has been employed and operationalized in previous studies, political commitment encompasses what governments say (expressed commitment), what policies they establish (institutional commitment) and what they invest (budgetary commitment), but each of these three dimensions of commitment have normative and evidence-based aspects that need to be considered in a conceptualization and empirical test of commitment.

### Determinants, effect modifiers and mediators of political commitment

To accurately draw inferences about the effect of political commitment on HIV outcomes, other factors that either determine both commitment and an outcome or modify the potential effect of commitment on the outcome should be accounted for. A growing body of literature, summarized in the following paragraphs, has examined why governments may or may not be committed to responding to HIV, which is consequential for understanding the effects of commitment on HIV outputs and outcomes.

Additionally, an understanding of the determinants of political commitment can assist researchers in thinking about how to build political commitment where it is lacking and an understanding of factors modifying or mediating the effect of political commitment can help identify intervention to improve outcomes at any level of commitment.

Studies have considered the following variables as either determinants or effect modifiers or mediators of political commitment: (1) the capacity of the state to respond to HIV (including economic resources) [68,69]; (2) the type of regime and governance [6-8,70,71]; (3) the heterogeneity of the population [25,45]; (4) the magnitude of the epidemic [3,11,45,69]; (5) the type of response to past epidemics [51,56]; (6) the intensity of international assistance [45,72]; and (7) the level of civil society involvement [73-76]. It is important for researchers to take such factors into account in their analyses of political commitment to respond to HIV because different countries have different baseline abilities to respond to this epidemic [11,12].

State capacity, which refers to the ability of the state to “implement decisions, mobilize resources, and enforce rules” [68,69], can determine the ability of its health sector to scale up interventions, absorb financial resources, and expand HIV services [68,69]. A state may be committed to political action, but lack the capacity to effectively carry out policy. Analyses of the effect of political commitment on outcomes should therefore examine in how far state capacity modifies or mediates commitment effects. Commonly used proxies for state capacity are economic development, government revenues and outlays, and physical infrastructure. For example, researchers have used as measures of state capacity to affect HIV outcomes, per capita gross national income, government expenditure on social services, secondary school enrolment, the ratio of healthcare providers to the population, the percentage of paved roads, and direct tax collection [68,69].

The type of regime of a country (democratic or authoritarian) has been hypothesized as a determinant of government commitment to fighting HIV [6-8,70,71]. Democratically elected governments should theoretically be more likely to commit to policies signalled as preferred by their constituents [36]. However, with critical exceptions like South Africa, in their respective analyses of HIV in Africa, Patterson (2006), Bor (2007) and Dionne (2011) find that democratic institutions do not predict strong AIDS policy responses [7,70]. Beyond regime type, good governance more broadly reflected in the quality of political, economic and administrative processes, such as fair elections, transparent management of economic resources, and citizens’ respect for the country’s institutions, has been hypothesized to improve political commitment to respond to HIV [8,71,77]. Using the World Bank governance indicators, Menon-Johannson (2005) finds evidence for a relationship between governance and HIV prevalence, whereas Reidpath and Allotey (2006) argue that causality in this relationship cannot be established without controlling for a range of structural factors correlated with governance, such as economic development and physical infrastructure [8,71,77]. Since regime type and governance may also influence government commitment, these variables should be accounted for in assessing the effects of government’s level of commitment to respond to HIV.

Characteristics of society that are outside the control of government also modify the effectiveness of commitment on outputs and outcomes. Ethnic diversity has been posited as a reason for low levels of public service provision (such as HIV treatment or prevention interventions), via constraints on intergroup collective action and decision making [78,79]. Boundary institutions, such as discriminatory employment or educational policies or group-differentiated personal law, can reinforce ethnic and religious divisions, which can hinder the ability of countries to commit to fighting HIV [25,45].

Higher levels of socio-economic inequality are also believed to depress the provision of public services through reduced social cohesion and increased disagreements over how scarce resources should be allocated [80,81]. Heterogeneity of societies can substantially impact how risk is constructed around HIV [47], as well as how resources are allocated to individuals affected by HIV infection [70]. Governments with more heterogeneous populations, both ethnically and economically, may therefore have a harder time committing to HIV policy or translating verbal commitments into action.

Given the varying types and magnitudes of epidemics (concentrated versus generalized; late versus early stage; high versus low HIV incidence), countries differ in their empirical urgency of addressing HIV. The magnitude of the epidemic, which may be equally attributable to underlying social and behavioural factors as to the political response, affects the saliency of the problem and thereby has the potential to increase public demand for action [6,7], which in turn should increase political commitment [36]. The challenge of implementing HIV interventions may also be greater for countries that have rapidly increasing HIV epidemics. In addition, more committed governments should also be more effective at reducing the epidemic, introducing simultaneity in the relationship between commitment and HIV outcomes. Since questions of the effects of political commitment usually cannot be investigated in controlled experiments, researchers need to consider observational study designs, such as natural experiments, controlled before/after studies, or regression discontinuity, which can provide rigorous counterfactuals for causal inferences in the absence of experimental intervention.

Researchers have further noted the path dependency of government responses to diseases: past commitment can affect future commitment [27,28][51][56]. Path dependency is a concept that has been used to explain the persistence of often ineffectual public policies in political science [29][54]. For example, Baldwin finds that past responses to contagious diseases conditioned countries’ responses to HIV, leading some European countries to adopt harsher, rights-constraining policies rather than more moderate, rights-enabling policies [56]. Likewise, Nunn finds that institutions developed in the early stages of Brazil’s response influenced subsequent aspects of its response and conditioned government commitment to responding to HIV [51]. Researchers should consider the legacy of existing institutions and how they condition commitment to HIV.

The contribution of the donor and non-governmental sector to a country’s political commitment must also be taken into account in order to avoid confounding government effort with non-governmental effort in addressing HIV. Non-governmental and governmental commitment may affect each other in complex ways. First, donor commitments tend to go towards countries that have signalled a political commitment to respond to HIV [45]. At the same time, donor contributions may replace domestic spending on HIV.

Second, numerous studies stress the role of social movements and civil society pressure in catalyzing greater government attention to HIV [23,73-76]. In some countries, however, civil society organizations have taken over functions in the HIV response that are traditionally considered to be state functions, and donor funds have increasingly bypassed the state, directly supporting civil society. Strong civil society engagement in the HIV response can therefore reduce the need for state commitment as the state delegated the response to non-state actors and may make the state appear less committed than it actually is [73]. Research on political commitment should investigate the effects of donor efforts and civil society responses on government commitment to fight HIV.

In sum, the factors that we have outlined (state capacity, governance, fractionalization and heterogeneity of the populace, the magnitude of disease, historical institutions, and non-governmental contributions) may act as determinants, effect modifiers or mediators of government commitment to respond to HIV. These factors should be accounted for in analyses seeking to explain the effect of political commitment on HIV-related outputs and outcomes.

### Outputs and outcomes of political commitment

**Figure 1:**
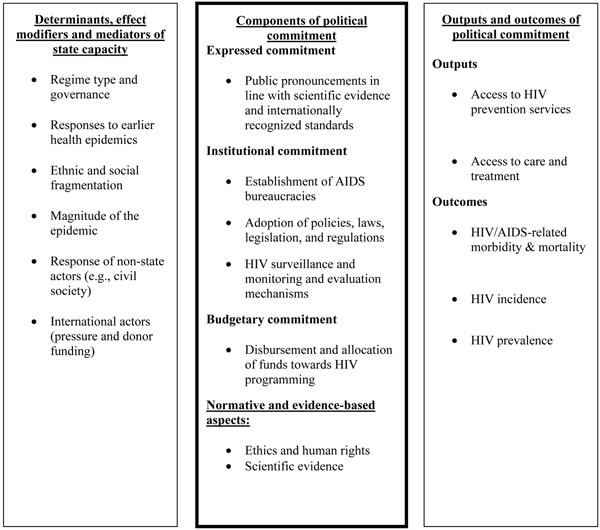
Framework for assessing political commitment to respond to HIV.

The extent to which a government politically commits to respond to HIV has important implications for how the epidemic develops and impacts those affected by the disease. An important distinction, which we reflect on in our conceptual framework, is between outputs and outcomes of political commitment to respond to HIV (Figure 1). Outputs are directly linked to a government’s institutional and budgetary commitment to responding to HIV, and outcomes are only linked to political commitment via their relationship with outputs. Examples of outputs that may be affected by political commitment are knowledge, attitudes and beliefs about the HIV epidemic, and HIV prevention, care and treatment coverage, whereas outcomes refer to HIV prevalence, incidence, morbidity and mortality. Political commitment is required to plan, set up and fund HIV interventions, so that intervention coverage is likely to be a function of commitment. Political commitment, expressed in politicians’ speeches and public discussions, can affect a population’s knowledge, attitudes and beliefs about the HIV epidemic, which in turn may influence behaviour and HIV health outcomes. Institutions and budgetary allocations can impact HIV outputs, such as access to treatment and prevention services, which in turn may affect outcomes.

Some studies measure commitment directly in terms of service delivery or the availability of ART and PMTCT therapies [11,12]. Yet, access to services is better viewed as an output of commitment rather than a measure of commitment itself.

## Discussion

### Applying the framework to assess the effect of political commitment on HIV outcomes

What is clear from this discussion is that political “commitment” to combat HIV is a complex construct made up of different dimensions and affected by different aspects of commitment. Although studies vary in their focus, a complete picture of a government’s political commitment necessitates accounting for each component of commitment. For example, governments may verbally commit to HIV, making public pronouncements for instrumental reasons (e.g., to attract donor funds), but fail to translate this rhetoric into action in the form of laws or investments in actual programmes. Conversely, governments may remain silent, but have all of the institutional infrastructure in place and invest resources towards HIV. It is also possible for governments to commit institutionally, but to underfund programmes, leading to incomplete policy implementation. Governments may invest in programmes but undermine them through public discourse or lack the institutional capacity to make use of funds. Judgements on the level of political commitment should therefore take simultaneous account of different components of commitment.

Even within a single country, political competition can lead to rival frames with political parties appealing to varying constructions of HIV risk and pressuring for different kinds of policy responses. In federal states, commitment may vary across relatively autonomous sub-national units making a uniform assessment of political commitment impossible. For federal states, the framework can be applied in microcosm to assess how differences in levels of commitment across sub-national units affect HIV outcomes. Alternatively, national policy or the average across states can be used to aggregate country responses.

Researchers must also use their discretion and be explicit in their choices concerning how to code government commitment according to its responsiveness to human rights and ethical standards (i.e., normative aspects) and the use of medical evidence in line with international standards (i.e., evidence-based aspects). In addition, background factors that affect the ability of the state to respond should be taken into account in assessing a country’s level of commitment. A country with weak state capacity (e.g., because of heterogeneity and fractionalization or a low resource base) may find it more difficult to translate commitment into an effective response.

Though it is tempting to attribute rapidly increasing or declining infection rates to political commitment, even if commitment is correctly specified for a given country, a number of challenges arise in causally linking commitment to HIV outcomes, particularly reduced incidence and HIV-related mortality [24]. Scholars should be aware that even when a country scores highly on its level of commitment to respond to HIV, its observed success in managing the epidemic may be causally related to something else (e.g., the efforts of civil society groups or a natural decline in the epidemic). Ideally, observational studies should employ strong approaches to identify causal effects using, for instance, natural experiments or sharp discontinuities in commitment levels.

**Figure 2:**
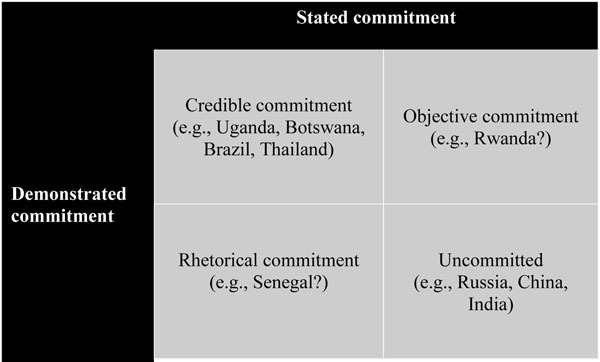
Combinations of commitment and country cases.

Because institutional and budgetary commitments are more concrete and objective than expressed commitments, which may be rhetorical, instrumental or symbolically driven, these components can be thought of as a form of “demonstrated” commitment rather than mere “stated” commitments. Countries can then be assessed on the degree to which their stated commitments corresponds with demonstrated commitments. To demonstrate this conceptualization of commitment, we can imagine a four-by-four table that would divide countries according to their levels of stated commitment and demonstrated commitment: (1) both stated and demonstrated commitment (credible commitment); (2) stated but not demonstrated commitment (rhetorical commitment); (3) demonstrated commitment without stated commitment (objective commitment); and (4) low stated and demonstrated commitment (uncommitted) (Figure 2).

Examples of countries that have credibly committed to HIV both in terms of actions and words could include Uganda, Botswana, Brazil and Thailand [11]. In these countries, institutional and budgetary commitments have been backed up by verbal commitments from the governments and have developed over time to a point where reversal of course is more difficult to achieve than a continuation. An example of a country with rhetorical commitment might be Senegal, which was praised early on by researchers for its openness to discussing HIV and the government’s explicit engagement with religious leaders in the response [82,83]. In terms of actual expenditures on HIV, however, Senegal’s budget remains modest and performance on outputs, such as ARV coverage for its small population of people living with HIV, low [11].

Rwanda, on the other hand, has been extremely effective at allocating resources for treatment and creating necessary institutions [11], but has issued few public statements highlighting HIV as a public health threat, leading some to question whether the country’s commitment to HIV is more strategic than genuine [84].

Finally, many countries might fall into the category of uncommitted, including Russia, India and China. These countries have not, or have only very recently, begun to publicly address HIV as a substantial public health threat and to build substantial institutional or budgetary commitments to respond to HIV [11]. For Russia and China, this is the case even though both countries have more state capacity, particularly in the form of financial resources, than many other developing countries to address this disease [11].

Bringing in normative and evidence-based dimensions to these examples could add additional nuances to the interpretations. Although generally interpreted as committed, Botswana’s routine opt-out testing policy has been criticized by international human rights observers [58], and Thailand’s lack of harm-reduction policies for injection drug users has been scorned [85]. Researchers have at various times questioned the evidence base of Uganda’s response, which committed to HIV prevention interventions at times when these interventions have lacked strong scientific support or were scientifically contested [48,62]. Using an objective measure of verbal commitment agnostic to contents, South Africa would be considered verbally committed, even though a large amount of the attention that past leaders have paid was to scientifically contested prevention and treatment strategies. Likewise, the Gambia's president, Alhaji Yahya Jammeh, has both verbally committed to his own herbal remedy for HIV and set up a parallel state-run treatment programme consisting of Islamic and traditional medicines [86].

These cases would have to be tested more systematically using available metrics (e.g., UNGASS and AIDS Program Effort Index indicators), discourse analysis of government speeches, and other appropriate methods. The type of commitment should also be matched with relevant outcomes, as in the case of Uganda, whose stated and demonstrated commitments have been oriented towards prevention, whereas Brazil and Botswana have emphasized treatment.

In spite of the challenges involved in measuring political commitment and coding countries accordingly, there are steps that researchers can take to assess the causal association between political commitment and HIV outcomes. Our examples are illustrations of how the framework can be applied in a more systematic manner.

As outlined in this paper, the steps that researchers may take to determine whether policy had an effect on HIV outcomes are the following:

1. Evaluate expressed commitment, including the frequency, timing and content of statements by key leadership.

2. Assess institutional commitment in terms of the presence of laws, policies, procedures and institutions addressing HIV prevention, care and treatment.

3. Calculate budgetary commitment in terms of both actual resources pledged and allocated towards HIV prevention, care and treatment.

4. Judge the alignment of the response with human rights, ethical standards, and scientific evidence, and decide whether to adopt a measure that defines commitment according to normative and evidence-based aspects of the response or a measure that is agnostic to these standards.

5. Account for state capacity with explicit consideration of international and non-governmental contributions.

6. Gauge performance on outputs and outcomes and alignment with the timing and type of policy response undertaken and, where possible, use strong observational quantitative study designs to identify causal effects.

7. Consider alternative explanations for outcomes (e.g., social and behavioural factors unrelated to political commitment).

## Conclusions

Assessing political commitment to respond to HIV is not a straightforward process. It is made complex by the fact that commitment has several dimensions (expressed, institutional and budgetary) and aspects (normative and evidence-based) along which a country can be judged. In general, better studies of commitment take into account more of these dimensions, but not every research question calls for evaluating each of these components. In addition, domestic political processes are influenced by a number of factors, which should be accounted for in assessing the relationship between commitment and HIV outcomes, because they confound, modify or mediate the relationship.

Despite these complexities, there are specific steps that researchers can take to accurately assess a country’s level of political commitment to respond to HIV and how this commitment ultimately influences HIV outcomes.

## Competing interests

The authors have no competing interests to declare.

## Authors' contributions

All authors contributed to the conception, design, writing and revision of this manuscript, and have given final approval of the version to be published.
